# Animal Models of Ischemic Stroke with Different Forms of Middle Cerebral Artery Occlusion

**DOI:** 10.3390/brainsci13071007

**Published:** 2023-06-29

**Authors:** Lang Zeng, Shengqi Hu, Lingcheng Zeng, Rudong Chen, Hua Li, Jiasheng Yu, Hongkuan Yang

**Affiliations:** Department of Neurosurgery, Tongji Hospital, Tongji Medical College, Huazhong University of Science and Technology, Wuhan 430074, China; gdzenglang@163.com (L.Z.); hsqhuaqi@163.com (S.H.); zenglingcheng923@hotmail.com (L.Z.); coney0522@163.com (R.C.); huali@hust.edu.cn (H.L.); yujiasheng2000@tjh.timu.edu.cn (J.Y.)

**Keywords:** ischemic stroke, middle cerebral artery occlusion model, thrombosis

## Abstract

Ischemic stroke is a common type of stroke that significantly affects human well-being and quality of life. In order to further characterize the pathophysiology of ischemic stroke and develop new treatment strategies, ischemic stroke models with controllable and consistent response to potential clinical treatments are urgently needed. The middle cerebral artery occlusion (MCAO) model is currently the most widely used animal model of ischemic stroke. This review discusses various methods for constructing the MCAO model and compares their advantages and disadvantages in order to provide better approaches for studying ischemic stroke.

## 1. Introduction

The term “stroke” (cerebrovascular disease or CVD) refers to a group of ailments affecting the blood vessels in the brain, including ischemic stroke, hemorrhagic stroke, and subarachnoid hemorrhage [1]. Ischemic stroke is the most common form of stroke, especially in the elderly, accounting for approximately 85% of all cases [2]. This condition is caused by the blockage of a cerebral blood vessel, which results in temporary or permanent brain damage. Ischemic stroke is a leading cause of death worldwide, accounting for about 9.6% of deaths, and is the third most common cause of death and disability in the United States [3]. Therefore, understanding the pathophysiology of ischemic stroke is crucial for developing safer and more effective treatment protocols. Medical researchers should systematically investigate the pathophysiological changes in ischemic CVD to establish a basis for treatment protocols, explore innovative prevention and treatment methods, and improve patient outcomes [4,5].

The middle cerebral artery occlusion (MCAO) model has been widely used as a valuable tool for studying focal ischemic stroke since its establishment in 1981. Tamuraet et al. [6] first demonstrated the permanent occlusion of the distal middle cerebral artery (MCA) through craniectomy. In 1986, Koizumi et al. developed a transient MCAO model in rats, which was later refined by Longa et al. The Koizumi method involves inserting a silicone-tipped nylon monofilament through the common carotid artery (CCA) and advancing it to the origin of the MCA through the internal carotid artery (ICA). On the other hand, the Longa method uses a similar intraluminal filament technique, but the filament is inserted through the external carotid artery (ECA) instead of the CCA [7]. In both the Koizumi and Longa methods, reperfusion is achieved by withdrawing the filament. However, the CCA is permanently ligated in the Koizumi method while reperfused in the Longa method. As a result, the Koizumi method can only be used in rodents with a well-developed circle of Willis, which allows for adequate blood flow in the MCA, whereas the Longa method can be used in any rodent [8,9].

Intracranial ischemia caused by ischemic CVD can be divided into focal ischemia and diffuse cerebral ischemia. The causes of intracranial ischemia involve MCA embolization, ICA or vertebral artery (VA) stenosis, occlusion or thrombosis, and cerebral artery spasm. Although traditional animal models have several limitations, they are still irreplaceable in specific research areas. Recently, some researchers have continued to improve the traditional models of intracranial ischemia and even tried to develop a novel model that has more promising outcomes [10,11,12]. For the purpose of this systematic review, the suggested PRISMA guidelines were adhered to [13].

The traditional model of intracranial ischemia has been developed over many years with significant advantages and limitations, and the most common type is the MCAO model. Previous studies have utilized various approaches, such as surgical clips, coils, and electrocoagulation after craniotomy, to occlude arteries in the brain. Other widely used methods also include the suture method, photochemical method, and thromboembolism [14]. Here, we describe a few more traditional models.

## 2. Intraluminal Filament Technique

The MCAO model is widely utilized in ischemic stroke research. Suture or filament occlusion of the MCA is a well-established method for inducing reproducible infarcts in the MCA region. This method does not require craniectomy and allows for reperfusion upon withdrawing the occluding filament, which mimics the therapeutic procedure of mechanical thrombectomy. As a result, this method has been increasingly applied to the animal modeling of stroke patients [7,15,16,17].

The advantage of this model is that the reperfusion time can be controlled accurately by removing the filament, thus achieving permanent or temporary occlusion. The ischemic penumbra is pronounced and does not sustain more injuries than the craniotomy procedure. However, its disadvantages include a lack of visibility, risk of subarachnoid hemorrhage, and inapplicability to thrombolytic studies. The intracranial filament method has been widely used in the study of reperfusion injury, ischemic penumbra, and the time window of thrombolytic therapy and can be used to simulate the removal of endovascular thrombosis [5].

## 3. Electrocoagulation

Both intraluminal filament and microsurgical direct MCAO using electrocoagulation are frequently used techniques for generating MCAO in mice [18]. This model is known to produce more consistent infarct sizes compared to the intraluminal occlusion model, but the resulting infarcts are typically smaller and may limit the assessment of neurobehavioral outcomes in stroke research. To address this limitation, researchers have modified the conventional electrocoagulation method to create a more stable and reproducible model of focal cerebral ischemia in mice that would result in larger infarct sizes and more profound neurobehavioral changes following cerebral ischemia [19].

Electrocoagulation is performed by exposing the MCA via craniotomy and burning the corresponding region of the MCA with electrodes to block the blood vessels and cause cortical or subcortical infarction within the supply area of the MCA [20].

High visibility and rapid disease development are two benefits of this irreversible acute cerebral ischemia animal model. However, this model requires craniotomy and may consequently result in surgical harm. Additionally, its application is limited by the requirement of adequate surgical skills. Moreover, since this approach only generates permanent blockage, it is not applicable to the research of thrombolytic therapy [14].

## 4. Photochemically Initiated Thrombosis

The photochemically initiated thrombosis (PIT) method was first established by Watson et al. and has been continuously optimized by researchers, such as in the stereotactic implantation of optical fibers to induce photochemical reactions in the cortex [21,22]. The PIT technique is a viable method for inducing cerebral infarction in rats through a reproducible thrombotic process, thereby providing a feasible animal model for studying ischemic stroke in humans.

Compared with traditional MCAO models, PIT can accurately locate the infarction foci and induce localized cortical damage. In addition, photochemically induced ischemia does not affect the internal capsule and striatum, nor the recovery of ischemic areas.

In small animals with thin skulls and good light permeability, PIT can create a penumbra while keeping the skull intact. Limited to the light region, the majority of the brain structure is not destroyed by controlling the severity of blood vessel damage through the modification of the plasma dye concentration, light intensity, and duration. The PIT model is appropriate for studying the mechanisms of acute phase interventions or a specific area of the brain, which is useful for focusing on lesion sites that are associated with behavioral alterations. In contrast, blood vessel damage results in vasogenic edema, which is fundamentally different from stroke in humans. Because the PIT model cannot induce severe functional deficiency in experimental animals, it is not suitable for the testing of neuroprotective medications. In addition, a craniotomy may partially compromise the integrity of intracranial structures, which renders the model less translatable to human research. Furthermore, it is also challenging to track changes in the circulation using this model. Endothelial cell destruction, blood–brain barrier damage, and vasogenic cerebral edema are possible complications that may not withstand extensive experimental observation and efficacy testing [21,23,24].

## 5. Homologous Blood Emboli

The homologous blood emboli approach induces thrombosis by directly injecting thrombin or introducing blood clots in the brain. The thromboembolism model was first established by Kudo et al. [25]. Some researchers occluded vessels by injecting a suspension of blood clot fragments into the common carotid or internal carotid arteries, while others used microcatheters to introduce blood clots directly into the beginning of the MCA [17].

This model addresses the problem of the inadequate clinical relevance of most models and better mimics the mechanism of human stroke. In addition, recombinant tissue plasminogen activators (rt-PAs) can be applied to this model to achieve recommunication, which is suitable for the research of new thrombolytic treatments. However, disadvantages of this model include the inability to accurately locate the clot and the tendency for blood clots to degrade and form small emboli that block small blood vessels and cause secondary embolism, thus resulting in variably sized, widely scattered, and unreproducible infarcts [26,27,28,29,30,31].

## 6. Novel Cerebral Ischemia Models

There have been ongoing efforts in improving the conventional models of cerebral ischemia through modifying the mechanisms of artery occlusion in order to improve the applicability of these models for studying ischemia in humans.

### 6.1. Ferric Chloride (FeCl_3_)-Induced Thrombus Formation

The limitations faced by traditional models of cerebral ischemia have been gradually overcome by the advancement in science and technology. In vivo multiphoton microscopy and laser speckle imaging have been increasingly used in stroke research due to their advantages in the real-time monitoring of the pathophysiological processes of cerebral ischemia. In addition, the traditional models of proximal occlusion cerebral ischemia are difficult to construct microscopically, and most models of distal occlusion cerebral ischemia are limited by the potential risk of injuries to the dura mater and periarterial cortex [32,33,34,35,36]. In order to address this issue, some researchers have suggested the topical application of FeCl_3_ within the MCA as a means of inducing cerebral ischemia in mice. FeCl_3_-induced thrombus formation has been extensively employed as an experimental model for both arterial and venous thromboses in a wide range of animal species, including rodents.

The induction of thrombosis through ferric chloride was initially described by Kurz et al. [37] in their development of an animal model of arterial thrombosis for assessing new antithrombotic agents. In this model, the application of FeCl_3_ to a blood vessel briefly prompts the formation of a thrombus that is morphologically reminiscent to those observed in humans. Although FeCl_3_ has been widely used to induce thrombosis, its role in inducing cerebral ischemia via the MCA has not been characterized. Consequently, a new experimental model of focal cerebral ischemia was developed, wherein FeCl_3_ is topically applied to the trunk of the distal MCA. In the modeling process, the cranial window of mice was opened by microsurgery, and filter paper soaked in FeCl_3_ of a certain concentration was placed on the surface of the main vessels of the distal MCA to induce thrombosis.

This study demonstrates that the FeCl_3_-induced thrombosis model exhibits practicality, non-traumatic characteristics, low mortality rates, and is well-suited for research utilizing intravital microscopy. In addition, this model offers a unique opportunity to conduct direct microscopic investigations of thrombolysis, an area of considerable clinical significance that has been inadequately explored experimentally.

FeCl_3_ application induces a small and reproducible infarct without causing significant brain swelling or high mortality. This procedure is also easy to perform microscopically and can be used in conjunction with live multiphoton microscopy. Furthermore, this approach causes minimal additional damage to brain tissue. However, the main drawback of this model is its low sensitivity to rt-PA. As a result, this model is not ideal for the study of neuroprotective drugs due to the suboptimal thrombolytic reperfusion effects of rt-PA.

### 6.2. Endothelial Injury

Injury to the vascular intima can cause cerebral ischemia, and the underpinning mechanism involves arterial endothelial cell damage by drugs or mechanical stimulation or the promotion of an increased level of oxidized low-density lipoprotein (LDL) and inflammatory cell infiltration, thereby initiating the proliferation and migration of vascular smooth muscle cells and ultimately resulting in artery stenosis and atherosclerotic plaque formation [38,39].

One of the most notable techniques for inducing endothelial injury is balloon expansion. This approach mimics the process of percutaneous coronary balloon angioplasty (PTCA), which causes endothelial cell injury and vascular smooth muscle rupture, followed by thrombosis, smooth muscle proliferation, and migration.

After inserting the arterial sheath into the femoral artery, the catheter is sent into the aortic arch under the guidance of digital subtraction angiography (DSA). A small quantity of contrast agent is then injected to visualize the bilateral carotid arteries. Once the catheter reaches the right carotid artery, it is withdrawn to allow the balloon to expand in the middle of the carotid artery and thereby cause damage to the vascular intima [40].

These methods are relatively simple and low-cost, but the formation of emboli is random and the emboli may easily dislodge and cause cerebrovascular accidents. It is precisely for this reason that few researchers have applied this method. The cerebral ischemia model established by intimal injury and stenosis is more reminiscent of the pathophysiological process of acute and chronic cerebral ischemia injury in humans. This model has irreplaceable advantages and is a relatively simple and minimally invasive operation. However, due to the unstable and inaccurate positioning of the thrombus, the success rate of thrombus formation is relatively low, leading to discrepancies in experimental results [41].

### 6.3. In Situ Thrombin Injection

With the maturity of stereotactic technology, some researchers proposed the in situ injection of thrombin to induce MCAO, which provides an effective way to more accurately locate the infarction, control the infarct area, improve model repeatability, and allow the real-time monitoring of the experimental process [42,43,44].

Animal mortality of this model is significantly lower than that of other models [28]. Rt-PA is the only thrombolytic agent approved for the treatment of stroke and has often been used to evaluate the clinical relevance of this model, which thus far has been completely consistent with the clinical data on rt-PA therapy. Therefore, this model has strong clinical relevance and is suitable for experiments with new thrombolytic drugs. In the study of thrombolysis using this model, researchers can intuitively grasp the obstruction and recanalization of blood vessels. However, the required craniotomy may cause certain damage to mice, and there is a certain possibility of the spontaneous rupture of the blood clots to form microthrombosis [42,43,44].

### 6.4. Model of Multifocal Cerebral Microinfarcts

Alzheimer’s disease (AD) is the leading cause of dementia, and current research indicates that vascular abnormalities in AD patients may increase the risk of cerebral microinfarcts, which affect about half of AD patients. Lecordier S et al. [45] used a novel technique to imitate multifocal microinfarcts in mice using sporadic microvascular occlusions in order to examine the effects of multifocal cerebral microinfarcts on the development of early AD-like pathology in young animals.

Briefly stated, mice were given 1.5% isoflurane in 1.5 l/min to induce sleep, and a feedback-controlled heating system was used to maintain their body temperatures between 36 and 37 °C. To expose the left CCA, as well as the ECA and the pterygopalatine artery (PPA), which were momentarily occluded using a microvascular clip under a surgical microscope, a midline neck incision was made. Then, using a 33G hypodermic needle, 2500 sterile, FITC-tagged microspheres of 20 m suspended in 100 µL of PBS were slowly injected into the CCA [46].

The effects of multifocal cerebral microinfarcts on the development of Alzheimer’s disease (AD) in male and female APP/PS1 mice were investigated in this work. The results showed that microinfarcts decreased A deposits in both sexes but caused more severe and long-lasting cognitive impairments in men than in women. Female mice recovered from the acute phase, whereas male mice underwent acute hypoperfusion followed by persistent hyperperfusion. Microinfarcts also induced elevated microglial activation and peripheral monocyte recruitment, which decreased A deposition, especially in females. In addition, male mice had an increased Dickkopf-1 (DKK1) expression, whereas female mice had a decreased expression. These findings demonstrate how microinfarcts, which are independent of A deposition, have a gender-specific impact on the evolution of AD pathogenesis.

## 7. Conclusions

Middle-aged and elderly individuals suffering from ischemic stroke often have a reduced quality of life and life expectancy. The most updated animal models are crucial for stroke research due to the constantly evolving treatment approaches for stroke patients. Therefore, more precise animal models are needed to replicate human conditions [47,48,49]. Mechanical occlusions caused by electrocoagulation, a filament, or a ligature have been employed in a number of animal stroke models. Injections of autologous or heterologous premade fibrin, blood clots including microemboli, or in situ clot formation using rose Bengal have all been utilized as models by other researchers. Although these latter models are more closely related to what occurs in stroke patients, they exhibit a high level of mortality, low reproducibility, and inconsistency in the number and location of infarcts, making statistical analysis of the data challenging [17].

The pathophysiological process of human stroke is quite complex. At present, most animal models of cerebral ischemia can simulate only one or several aspects of the mechanism of stroke, and there is no universally applicable animal model of cerebral ischemia. However, the authors believe that as long as we understand the advantages and disadvantages of different cerebral ischemia models, select animal models that are appropriate for the research direction, and constantly improve the models by incorporating new techniques, we can better simulate complications and control physiological-influencing factors. In this way, basic research can finally be integrated with clinical practice, resulting in breakthroughs in stroke research and qualitative changes to the lives of millions of stroke patients.

## Abbreviations

MCAO: Middle cerebral artery occlusion, CVD: cerebrovascular disease, MCA: middle cerebral artery, CCA: common carotid artery, ICA: internal carotid artery, ECA: external carotid artery, PPA: pterygopalatine artery, VA: vertebral artery, FeCl_3:_ ferric chloride, LDL: low-density lipoprotein, PTCA: percutaneous coronary balloon angioplasty, DSA: digital subtraction angiography, AD: Alzheimer’s disease, PBS: phosphate-buffered saline.
